# Supplementary material for: CerS6-dependent ceramide synthesis in hypothalamic neurons promotes ER/mitochondrial stress and impairs glucose homeostasis in obese mice
Source: Nat Commun. 2023 Nov 29;14:7824. doi: 10.1038/s41467-023-42595-7 (PMC10684560; doi:10.1038/s41467-023-42595-7)
Supplement: Supplementary file 1 — Supplementary Information [file 41467_2023_42595_MOESM1_ESM.pdf]

Supplementary Information

CerS6-dependent ceramide synthesis in hypothalamic neurons promotes ER/mitochondrial stress and impairs glucose homeostasis in obese mice

Philipp Hammerschmidt, Sophie M. Steculorum, Cécile L. Bandet, Almudena Del Río-Martín, Lukas Steuernagel, Vivien Kohlhaas, Marvin Feldmann, Luis Varela, Adam Majcher, Marta Quatorze Correia, Rhena F. U. Klar, Corinna A. Bauder, Ecem Kaya, Marta Porniece-Kumar, Nasim Biglari, Anna Sieben, Tamas L. Horvath, Thorsten Hornemann, Susanne Brodesser, Jens C. Brüning

Supplementary Figure 1 (Related to Figure 1) Hammerschmidt et al., 2023

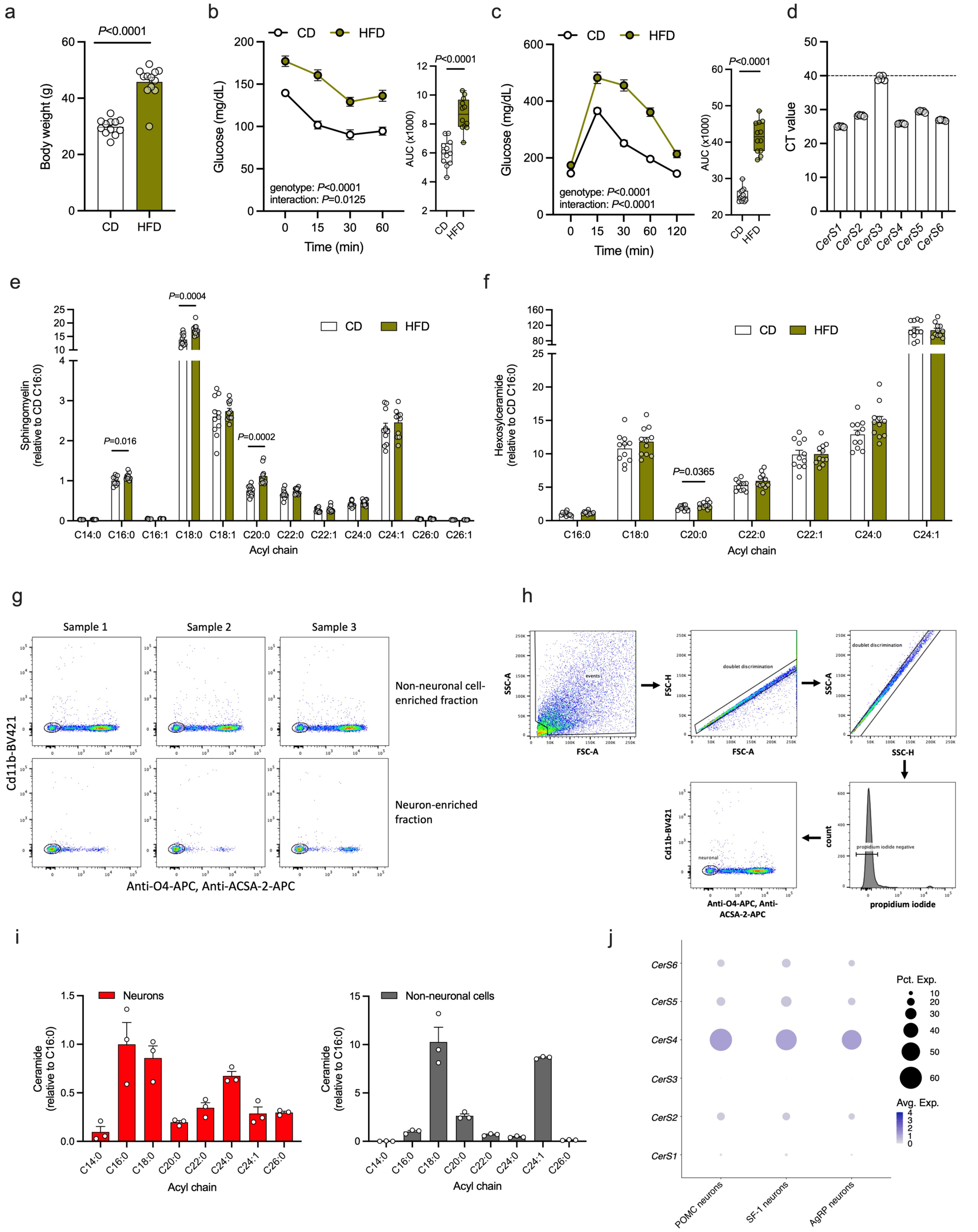

Supplementary Fig. 1: Metabolic characterization, sphingolipid levels, and *CerS* expression in C57BL/6N mice fed a control or high-fat diet. (Related to Fig. 1)

a-**c**, Metabolic characterization of C57BL/6N male mice used for analysis of CerS6 protein expression and sphingolipid content in hypothalamus homogenates. Mice fed *ad libitum* with a control diet (CD) or high-fat diet (HFD) for 16 weeks. **a**, Body weight of mice at 20 weeks of age when sacrificed for organ collection (n=12 mice/group). **b**, Insulin tolerance test at 16 weeks of age and area under the curve (AUC) for each mouse (n=12 mice/group). **c**, Glucose tolerance test following a 6 hr fasting period at 17 weeks of age and AUCs (n=12 mice/group). **d**, Cycle threshold (CT) values of qPCR analysis in hypothalamus homogenates of misty control mice using TaqMan probes targeting the different *CerS* mRNAs. Dashed line indicates detection threshold at 40 cycles (n=5 mice). **e**, **f**, Lipidomic analysis of sphingomyelin (**e**) and hexosylceramide species (**f**) in hypothalamus homogenates of CD- and HFD-fed mice relative to the respective $C_{16:0}$ sphingolipid species in CD-fed mice (n=11 mice/group). **g**, Flow cytometry-based analysis for non-neuronal cell markers CD11b (microglia, y axis), O4 (oligodendro-cytes, x axis), and ACSA-2 (astrocytes, x axis) in non-neuronal cell- (upper row) and neuron-enriched cell fractions (lower row) isolated from the hypothalamus of three normal chow-fed mice (Samples 1-3), respectively. FACS plot only demonstrates cell type purity but has no and requires no statistical analysis. **h**, Representative dot plots illustrate the gating strategy used to verify the neuronal and non-neuronal fractions shown in **g**. After doublet discrimination (FSC-H vs. FSC-A, SSC-A vs. SSC-H), dead cells were excluded based on propidium iodide fluorescence. Non-neuronal cells were excluded using CD11b, O4, and ACSA-2 markers. **i**, Lipidomic analysis of ceramides in the neuron-enriched cell fraction (red, Neurons) and non-neuronal cell-enriched fraction (grey, Non-neuronal cells) (n=3 mice). **j**, Dot plots of *CerS1-6* expression in POMC-, SF-1-, and AgRP-expressing neurons according to single-cell sequencing results harmonized in HypoMap. Data in **a**, **d**-**f**, **i**, and longitudinal data in **b**, **c** are represented as mean values ±SEM. Boxplots indicate median ±min/max and include data points of individual mice entering the analysis. *P* values calculated using two-tailed unpaired Student's *t*-test (**a**, **e**, **f**, AUCs in **b**, **c**) or two-way RM ANOVA (longitudinal analysis in **b**, **c**). Source data and further details of statistical analyses are provided as a Source Data file.

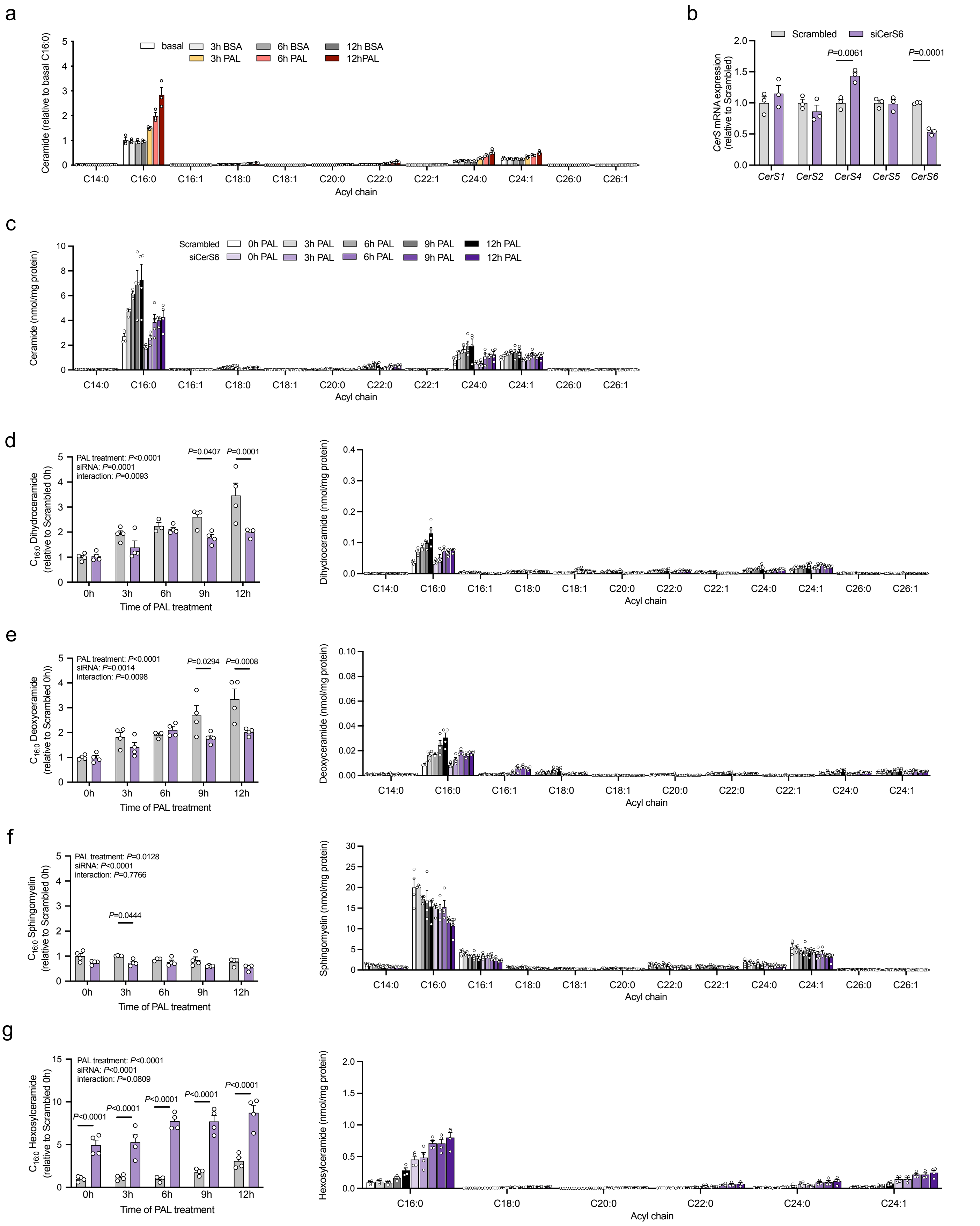
a
Ceramide (relative to basal C16:0)
basal
3h BSA
3h PAL
6h BSA
6h PAL
12h BSA
12hPAL
C14:0 C16:0 C16:1 C18:0 C18:1 C20:0 C22:0 C22:1 C24:0 C24:1 C26:0 C26:1
Acyl chain
b
Scrambled
siCerS6
CerS mRNA expression (relative to Scrambled)
P=0.0061
P=0.0001
CerS1 CerS2 CerS4 CerS5 CerS6
c
Scrambled
siCerS6
0h PAL
3h PAL
6h PAL
9h PAL
12h PAL
Ceramide (nmol/mg protein)
Acyl chain
d
PAL treatment: P<0.0001
siRNA: P=0.0001
interaction: P=0.0093
P=0.0407
P=0.0001
C16:0 Dihydroceramide (relative to Scrambled 0h)
Time of PAL treatment
Dihydroceramide (nmol/mg protein)
Acyl chain
e
PAL treatment: P<0.0001
siRNA: P=0.0014
interaction: P=0.0098
P=0.0294
P=0.0008
C16:0 Deoxyceramide (relative to Scrambled 0h))
Time of PAL treatment
Deoxyceramide (nmol/mg protein)
Acyl chain
f
PAL treatment: P=0.0128
siRNA: P<0.0001
interaction: P=0.7766
P=0.0444
C16:0 Sphingomyelin (relative to Scrambled 0h)
Time of PAL treatment
Sphingomyelin (nmol/mg protein)
Acyl chain
g
PAL treatment: P<0.0001
siRNA: P<0.0001
interaction: P=0.0809
P<0.0001
P<0.0001
P<0.0001
P<0.0001
P<0.0001
C16:0 Hexosylceramide (relative to Scrambled 0h)
Time of PAL treatment
Hexosylceramide (nmol/mg protein)
Acyl chain

Supplementary Fig. 2: CerS6 in mHypoE-N43/5 cells. (Related to Fig. 2)

a, Relative ceramide levels in N43/5 cells that were either left untreated (basal) or exposed to palmitate (PAL, 500 µM) or BSA for the time indicated (n=3 independent cultures). Values are expressed relative to the $C_{16:0}$ ceramide content at basal conditions. **b**, Relative mRNA expression of *CerS1-6* in N43/5 cells treated with siCerS6 (purple) or scrambled (grey, n=3 independent cultures with 3 replicate dishes/experiment). **c**, Absolute ceramide levels in siCerS6- or scrambled treated N43/5 cells incubated with PAL for the time indicated in hours (h) (n=4 independent cultures). **d**-**g**, $C_{16:0}$ sphingolipid species relative to levels at 0h BSA (left) and absolute levels (right) of dihydroceramides (**d**), deoxyceramides (**e**), sphingomyelins (**f**), and hexosylceramides (**g**) (n=4 independent cultures). Data are represented as mean values ±SEM. *P* values calculated using two-tailed unpaired Student's *t*-test (**b**) or two-way ANOVA and Bonferroni's multiple comparison test ($C_{16:0}$ sphingolipid levels in **d**-**g**). Source data and further details of statistical analyses are provided as a Source Data file.

Supplementary Figure 3 (Related to Figure 3) Hammerschmidt et al., 2023

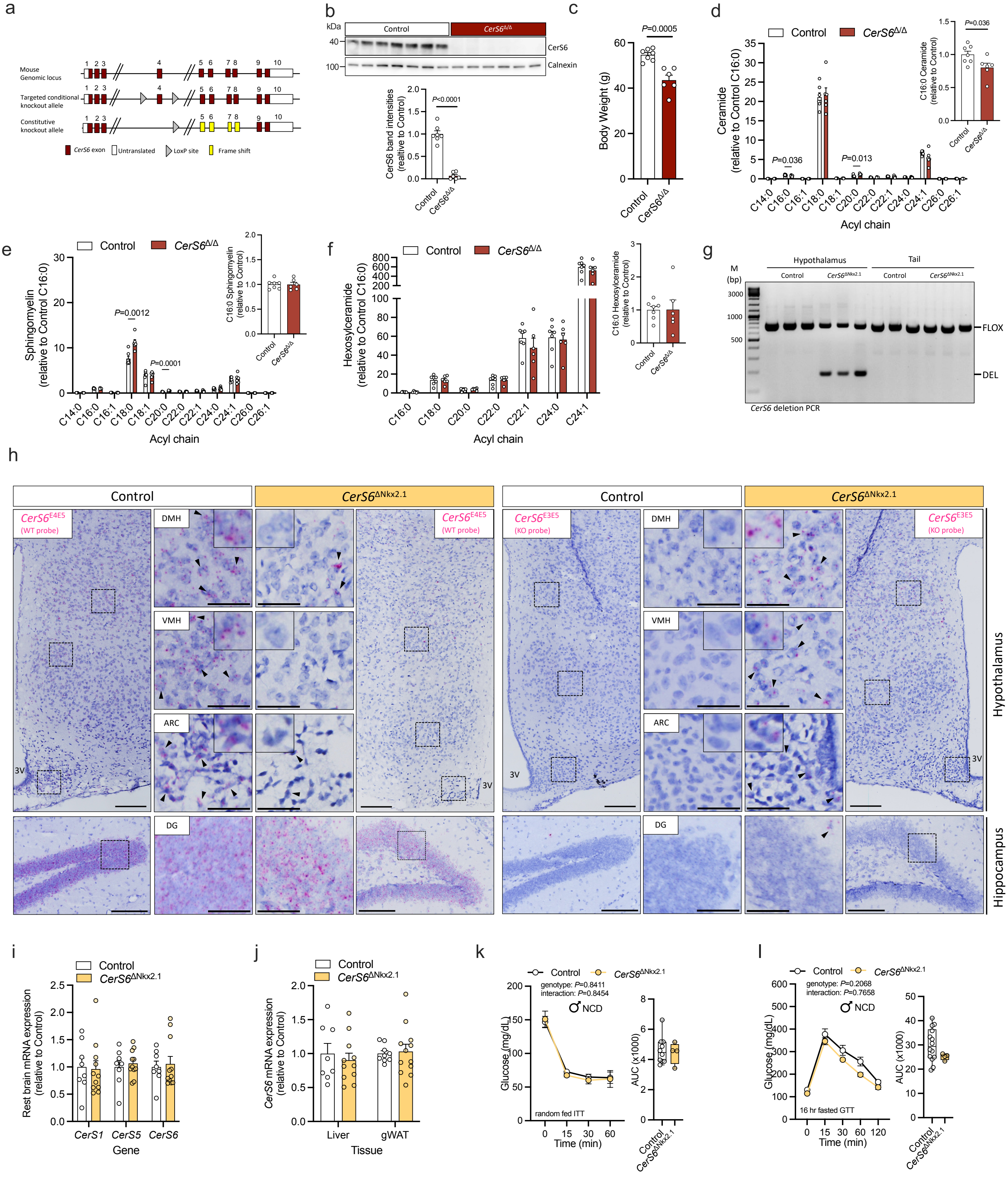

Supplementary Fig. 3: CerS6 deficiency in the hypothalamus and in hypothalamic neurons of mice. Related to Fig. 3

a, Simplified schematic illustration detailing deletion of exon 4 of the *CerS6* gene via Cre-loxP-mediated DNA recombination. **b**, Immunoblot analysis of CerS6 in hypothalamus homogenates of HFD-fed control (white) and HFD-fed conventional CerS6 knockout male mice ($CerS6^{\Delta/\Delta}$, red); CerS6 band intensities normalized for Calnexin and expressed as relative to control (n=7vs.6 mice). Uncropped blots in Source Data. **c**, Body weight of mice when sacrificed for organ collection (n=7vs.6 mice). **d**-**f**, Levels of ceramides (**d**), sphingomyelins (**e**), and hexosylceramides (**f**) in the hypothalamus of HFD-fed control and $CerS6^{\Delta/\Delta}$ mice relative to the respective $C_{16:0}$ sphingolipid species in controls (n=7vs.6 mice). Relative levels of $C_{16:0}$ ceramide, sphingomyelin, or hexosylceramide are highlighted separately. **g**, PCR analysis on genomic DNA for exon 4 deletion of *CerS6* in extracts of the hypothalamus and tail of $CerS6^{\Delta Nkx2.1}$ mice and controls (n=3 mice/group). **h**, Brightfield images of BaseScope in situ hybridization using a probe targeting the exon junction 4/5 in the *CerS6* transcript ($CerS6^{E4E5}$, red), illustrating expression of full-length *CerS6* (left), or the exon junction 3/5 ($CerS6^{E3E5}$, red), illustrating expression of the recombined exon 4-deficient transcript of *CerS6* (right) in the hypothalamus (top) and hippocampus (bottom) of $CerS6^{\Delta Nkx2.1}$ mice (orange) and controls (white); zoom images depict the dorsomedial nucleus (DMH), ventromedial nucleus (VMH), arcuate nucleus (ARC) of the hypothalamus, and the dentate gyrus (DG) of the hippocampus; blue denotes nuclei stained by haematoxylin; dashed squares indicate zoom range; black arrows depict cells positive for the respective *CerS*6 transcript; 3V, third ventricle; scale bars: 150 µm for overview images, 50 µm for zoom images. **i**, Relative mRNA expression of *CerS1*, *CerS5*, and *CerS6* in brain homogenates excluding the hypothalamus analyzed by qPCR in HFD-fed control and $CerS6^{\Delta Nkx2.1}$ mice (n=8vs.11 mice). **j**, Relative mRNA expression of *CerS6* in liver (n=8vs.11 mice) and gonadal white adipose tissue (gWAT) of control and $CerS6^{\Delta Nkx2.1}$ mice (n=9vs.12 mice). **k**, **l**, Insulin tolerance test (ITT) in random-fed mice (**k**, n=10vs.4 mice) and glucose tolerance test (GTT) after a 16 hr fasting period (**l**, n=14vs.4 mice) in NCD-fed controls and $CerS6^{\Delta Nkx2.1}$ mice. All data obtained from male mice. Data in b-j and longitudinal data in k, l are represented as mean values ±SEM. Boxplots indicate median ±min/max and include data points of individual mice entering the analysis. *P* values calculated using two-tailed unpaired Student's *t*-test (**b-e**) and two-way RM ANOVA (longitudinal analysis in **k**, **l**). Source data and further details of statistical analyses are provided as a Source Data file.

Supplementary Figure 4 (Related to Figure 3) Hammerschmidt et al., 2023

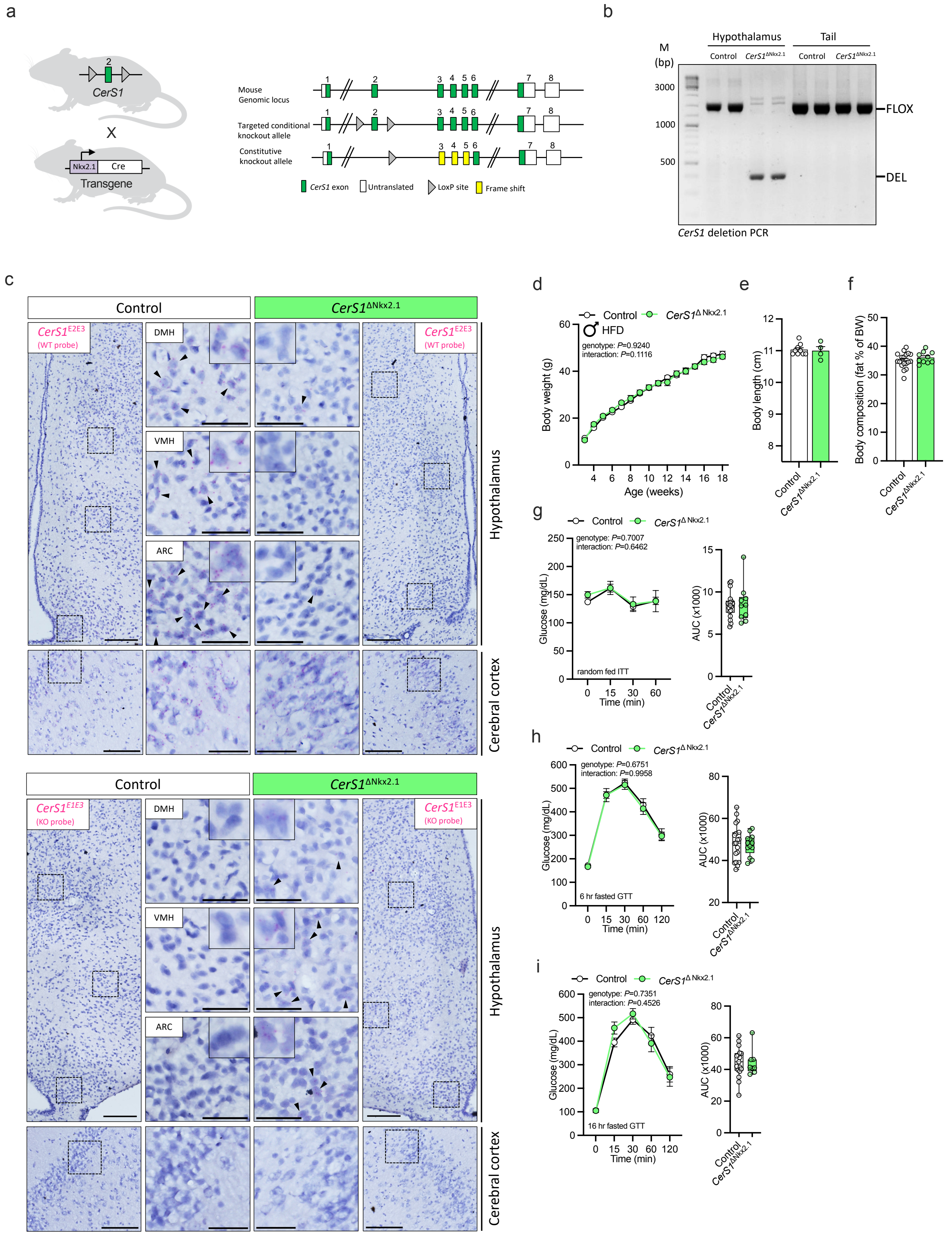

Supplementary Fig. 4: Depletion of CerS1 in hypothalamic neurons does not affect glucose metabolism in obesity. Related to Fig. 3

a, Left: *CerS1*$^{fl/fl}$ mice in which exon 2 of *CerS1* (green) is flanked by loxP sites (grey triangles) were bred to mice with transgenic Cre expression (white) under control of the Nkx2.1 promoter (purple). Right: Excision of exon 2 in *CerS1* leads to a frameshift in the downstream exons 3-5, preventing translation of the functional domain responsible for ceramide synthesis. *CerS1*$^{fl/fl}$ mice expressing Nkx2.1-Cre (*CerS1*$^{\Delta Nkx2.1}$, bright green) and Cre-negative *CerS1*$^{fl/fl}$ littermates (Control, white) were used for analysis. **b**, PCR analysis on genomic DNA for exon 2 deletion of *CerS1* in extracts of the hypothalamus and tail of *CerS1*$^{\Delta Nkx2.1}$ mice and controls (n=2 mice/group). **c,** Brightfield images of in situ hybridization using a BaseScope probe targeting the exon junction 2/3 in the *CerS1* transcript (*CerS1*^{E2E3}, red), illustrating expression of full-length *CerS1* (top), or a probe targeting the exon junction 1/3 (*CerS1*^{E1E3}, red), illustrating expression of the recombined exon 2-deficient transcript of *CerS1* (bottom) in the hypothalamus (top) and cerebral cortex (bottom) of *CerS1*$^{\Delta Nkx2.1}$ mice and controls; zoom images depict the dorsomedial nucleus (DMH), ventromedial nucleus (VMH), and arcuate nucleus (ARC) of the hypothalamus; blue denotes nuclei stained by haematoxylin; dashed squares indicate zoom range; black arrows depict cells positive for the respective *CerS1* transcript; 3V, third ventricle; scale bars: 150 µm for overview images, 50 µm for zoom images. **d**, Body weight development of male mice during HFD feeding (n=8-19 mice/group and week), **e**, Body length (n=9vs.4 mice), and **f**, body fat content relative to body weight, measured by nuclear magnetic resonance (n=18vs.10 mice). **g**, Insulin tolerance test and area under the curve (AUC) for each mouse (n=18vs.10 mice), **h**, glucose tolerance test following a 6 hr fasting period and AUCs (n=19vs.13 mice), and **i**, glucose tolerance test following a 16 hr fasting period and AUCs (n=17vs.8 mice)**.** All data obtained from male mice. Data in **d**-**f** and longitudinal data in **g**-**i** are represented as mean values ±SEM. Boxplots indicate median ±min/max and include data points of individual mice entering the analysis. *P* values calculated using a mixed-effects model (**d**) or two-way RM ANOVA (longitudinal analysis in **g**-**i**). Source data and further details of statistical analyses are provided as a Source Data file. Created with BioRender.com.

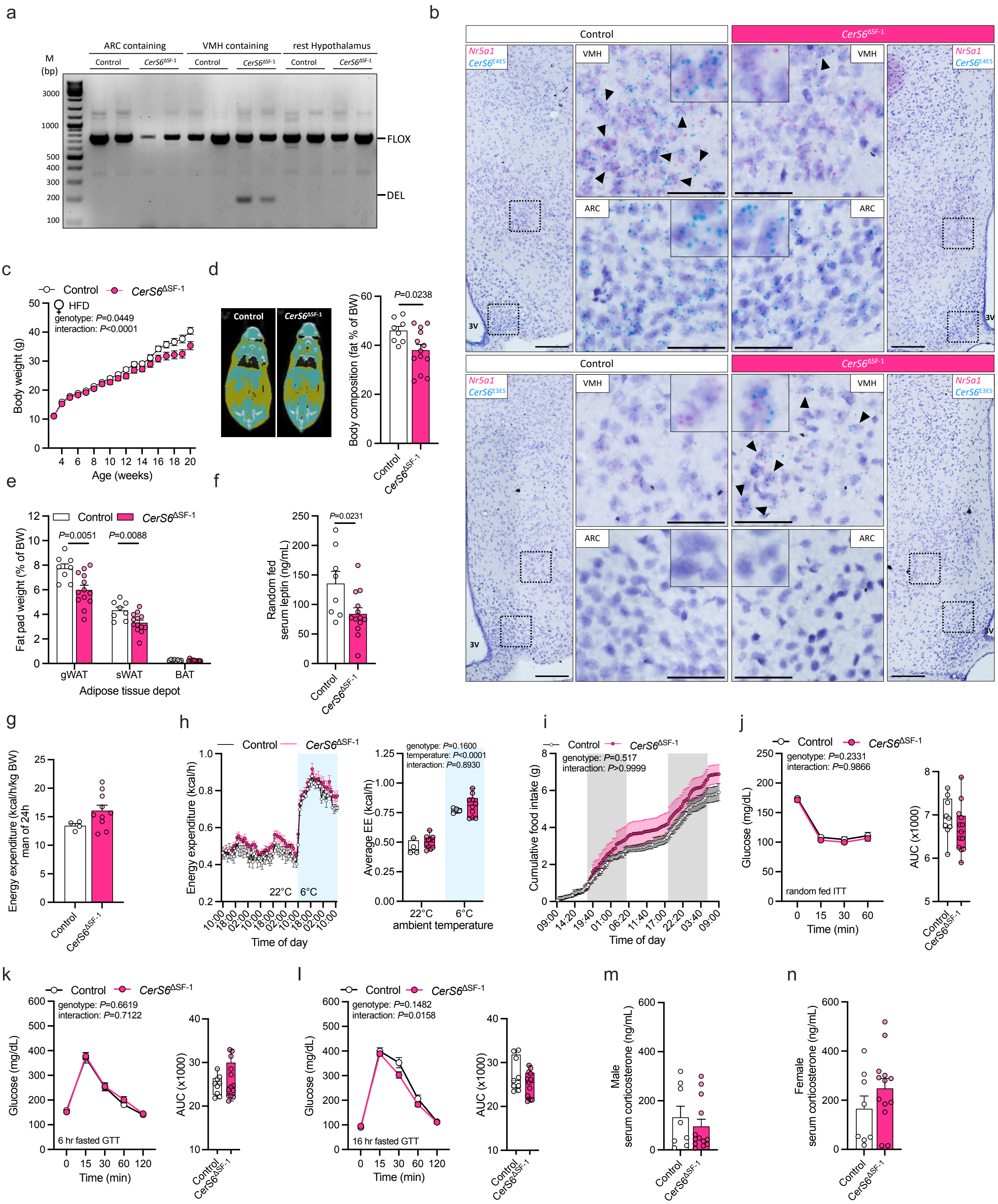
a
ARC containing
VMH containing
rest Hypothalamus
Control
CerS6ΔSF-1
M (bp)
3000
1000
500
400
300
200
100
FLOX
DEL
b
Control
CerS6ΔSF-1
Nr5a1
CerS6E4E5
CerS6E3E5
VMH
ARC
3V
c
Control
CerS6ΔSF-1
HFD
genotype: P=0.0449
interaction: P<0.0001
Body weight (g)
Age (weeks)
d
Body composition (fat % of BW)
P=0.0238
e
Fat pad weight (% of BW)
P=0.0051
P=0.0088
gWAT
sWAT
BAT
Adipose tissue depot
f
Random fed serum leptin (ng/mL)
P=0.0231
g
Energy expenditure (kcal/h/kg BW) man of 24h
h
Energy expenditure (kcal/h)
Time of day
22°C
6°C
genotype: P=0.1600
temperature: P<0.0001
interaction: P=0.8930
Average EE (kcal/h)
ambient temperature
i
Cumulative food intake (g)
genotype: P=0.517
interaction: P>0.9999
Time of day
j
genotype: P=0.2331
interaction: P=0.9866
Glucose (mg/dL)
random fed ITT
Time (min)
AUC (x1000)
k
genotype: P=0.6619
interaction: P=0.7122
6 hr fasted GTT
l
genotype: P=0.1482
interaction: P=0.0158
16 hr fasted GTT
m
Male serum corticosterone (ng/mL)
n
Female serum corticosterone (ng/mL)

Supplementary Fig. 5: Depletion of CerS6 in SF-1 neurons reduces adiposity in HFD-fed female mice. Related to Fig. 4

a, PCR analysis on genomic DNA for exon 4 deletion of *CerS6* in ARC- and VMH-containing tissue samples and the rest of the hypothalamus of control and $CerS6^{\Delta SF\text{-}1}$ mice (n=2 mice/group). **b**, Brightfield images of BaseScope duplex in situ hybridization using probes targeting *Nr5a1* (encoding SF-1, red) and the exon junction 4/5 in the *CerS6* transcript ($CerS6^{E4E5}$), illustrating expression of full-length *CerS6* (bright blue, top), or the exon junction 3/5 ($CerS6^{E3E5}$), illustrating expression of the recombined exon 4-deficient transcript of *CerS6* (bright blue, bottom) in the hypothalamus of $CerS6^{\Delta SF\text{-}1}$ mice (pink) and controls (white); zoom images depict the ventromedial nucleus (VMH) and arcuate nucleus (ARC) of the hypothalamus; blue denotes nuclei stained by haematoxylin; dashed squares indicate zoom range; black arrows depict cells coexpressing *Nr5a1* and the respective *CerS*6 transcript; 3V: third ventricle; scale bars: 150 µm for overview images, 50 µm for zoom images. **c-l**, Analysis of HFD-fed control (white) and $CerS6^{\Delta SF\text{-}1}$ female mice (pink). **c**, Body weight development of mice during HFD feeding (n=9vs.13 mice/weeks 3-13, n=8vs.13 mice/weeks 14-20). **d,** CT scans (yellow, fat tissue; blue, non-adipose soft tissue) and quantification of body fat content relative to body weight (n=8vs.13 mice). **e,** Fat-pad weights of gonadal white adipose tissue (gWAT, n=8vs.13 mice), subcutaneous WAT (sWAT, n=8vs.13), and brown adipose tissue (BAT, n=8vs.12) relative to body weight. **f,** Serum leptin levels of random-fed mice (n=8vs.13 mice). **g**, Average energy expenditure over 24 hr normalized for body weight (n=4vs.10 mice). **h**, Absolute energy expenditure (EE) at 22°C ambient temperature and upon exposure to 6°C, over time (left) and in average (right, n=4vs10 mice). **i**, Cumulative food intake over 48 hr (n=4vs.10 mice; grey background indicates dark phase). **j,** Insulin tolerance test and area under the curve (AUC) for each mouse (n=9vs.13 mice). **k, l,** Glucose tolerance test following a 6 hr (**k**) or 16 hr (**l**) fasting period and AUCs (n=9vs.13 mice). **m**, **n**, Serum corticosterone levels in random-fed male (**m**) and female (**n**) mice (n=8vs.13 mice/sex). All data in **c-l** obtained from female mice. Data in **c-g, m, n,** and longitudinal data in **h-l** are represented as mean values ±SEM. Boxplots indicate median ±min/max and include data points of individual mice entering the analysis. *P* values calculated using two-tailed unpaired Student's *t*-test (**d-f**), two-way ANOVA and Bonferroni's multiple comparison test (Average EE in **h**), two-way RM ANOVA (longitudinal analysis in **j-l**), or a mixed-effects model (**c**). Source data and further details of statistical analyses are provided as a Source Data file.

Supplementary Figure 6 (Related to Figure 5) Hammerschmidt et al., 2023

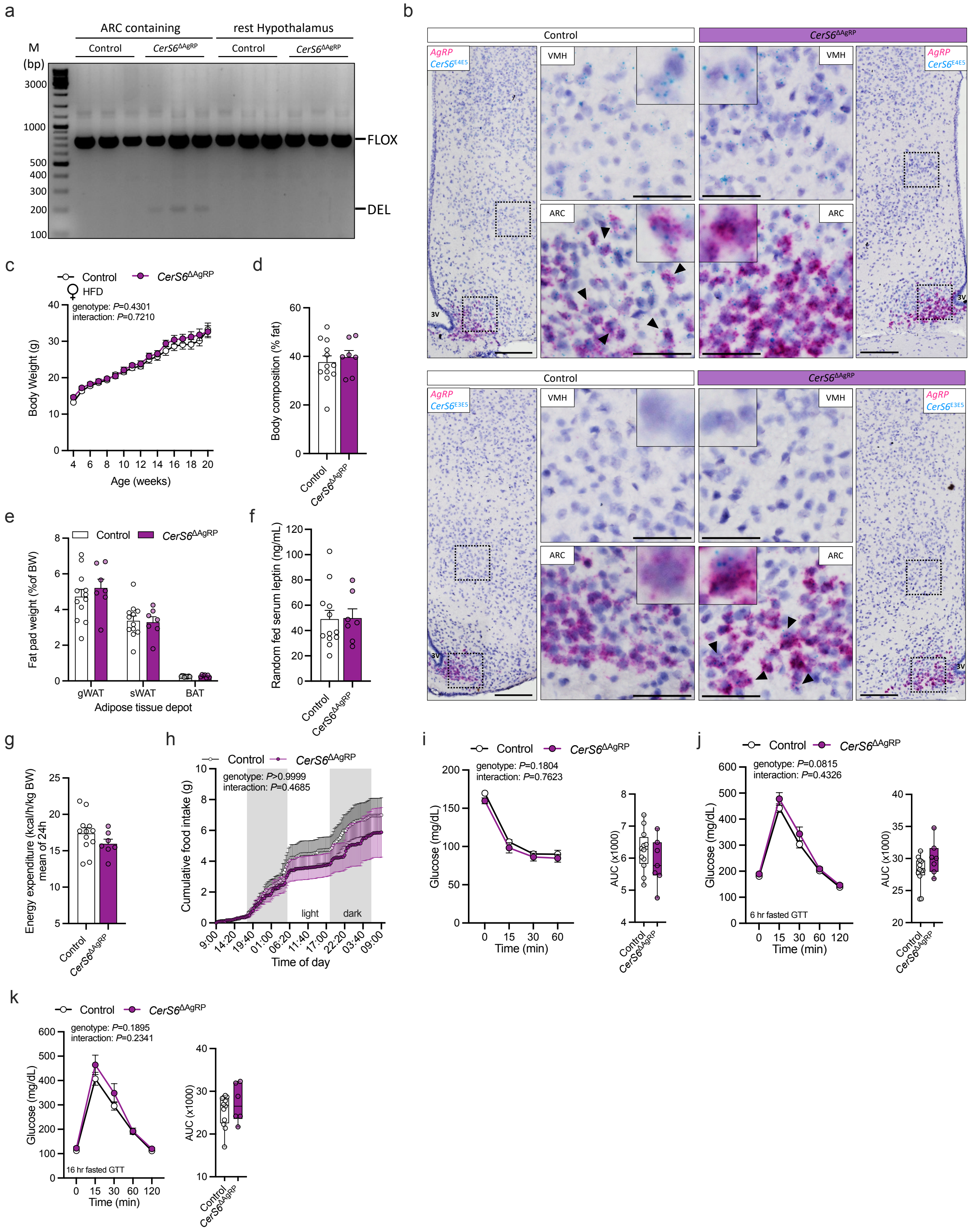

Supplementary Fig. 6: Depletion of CerS6 in AgRP neurons does not affect glucose and energy metabolism in HFD-fed female mice. Related to Fig. 5

a, PCR analysis on genomic DNA for exon 4 deletion of *CerS6* in ARC-containing tissue samples and the rest of the hypothalamus of control and $CerS6^{\Delta AgRP}$ mice (n=3 mice/group). **b**, Brightfield images of BaseScope duplex in situ hybridization using probes targeting *Agrp* (red) and the exon junction 4/5 in the *CerS6* transcript ($CerS6^{E4E5}$), illustrating expression of full-length *CerS6* (bright blue, top), or the exon junction 3/5 ($CerS6^{E3E5}$), illustrating expression of the recombined exon 4-deficient transcript of *CerS6* (bright blue, bottom) in the hypothalamus of $CerS6^{\Delta AgRP}$ mice (purple) and controls (white); zoom images depict the ventromedial nucleus (VMH) and arcuate nucleus (ARC) of the hypothalamus; blue denotes nuclei stained by haematoxylin; dashed squares indicate zoom range; black arrows depict cells coexpressing *Agrp* and the respective *CerS*6 transcript; 3V: third ventricle; scale bars: 150 µm for overview images, 50 µm for zoom images. **c-k**, Analysis of HFD-fed control (white) and $CerS6^{\Delta AgRP}$ (purple) female mice. **c**, Body weight development of mice during HFD feeding (n=3-12 mice/group and week). **d**, Body fat content relative to body weight measured by micro-CT analysis (n=12vs.7 mice). **e**, Fat-pad weights of gonadal white adipose tissue (gWAT), subcutaneous WAT (sWAT), and brown adipose tissue (BAT) relative to body weight (n=12vs.7 mice). **f,** Serum leptin levels of random-fed mice (n=12vs.7 mice). **g**, Average energy expenditure over 24 hr normalized for body weight (n=12vs.7 mice). **h,** Cumulative food intake over 48 hr (n=12vs.7 mice; grey background indicates dark phase). **i,** Insulin tolerance test and area under the curve (AUC) for each mouse (n=12vs.7 mice), **j,** glucose tolerance test following a 6 hr fasting period and AUCs (n=12vs.7 mice), and **k,** glucose tolerance test following a 16 hr fasting period and AUCs (n=12vs.6 mice). All data in (**c**-**k**) obtained from female mice. Data in **c-g**, and longitudinal data in **i-k** are represented as mean values ±SEM. Boxplots indicate median ±min/max and include data points of individual mice entering the analysis. *P* values calculated using two-way RM ANOVA (longitudinal analysis in **h**, **i-k**) or a mixed-effects model (**c**). Source data and further details of statistical analyses are provided as a Source Data file.

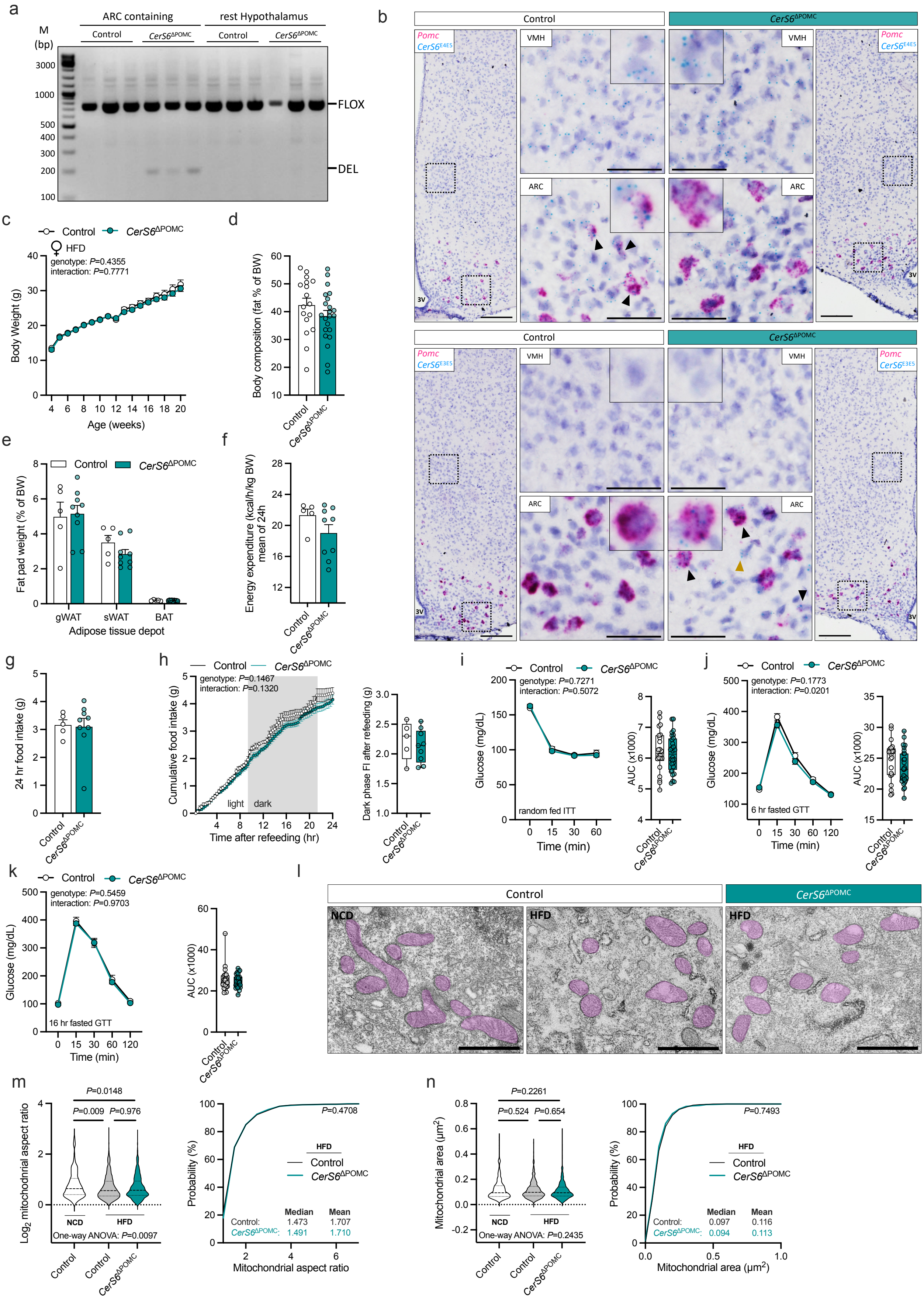
a
ARC containing
rest Hypothalamus
Control
CerS6ΔPOMC
M (bp)
FLOX
DEL
b
Control
CerS6ΔPOMC
Pomc
CerS6E4E5
VMH
ARC
3V
c
Control
CerS6ΔPOMC
HFD
genotype: P=0.4355
interaction: P=0.7771
Body Weight (g)
Age (weeks)
d
Body composition (fat % of BW)
e
Fat pad weight (% of BW)
gWAT
sWAT
BAT
Adipose tissue depot
f
Energy expenditure (kcal/h/kg BW) mean of 24h
g
24 hr food intake (g)
h
genotype: P=0.1467
interaction: P=0.1320
Cumulative food intake (g)
light
dark
Time after refeeding (hr)
Dark phase FI after refeeding (g)
i
genotype: P=0.7271
interaction: P=0.5072
Glucose (mg/dL)
random fed ITT
Time (min)
AUC (x1000)
j
genotype: P=0.1773
interaction: P=0.0201
6 hr fasted GTT
k
genotype: P=0.5459
interaction: P=0.9703
16 hr fasted GTT
l
NCD
HFD
m
P=0.0148
P=0.009
P=0.976
Log2 mitochondrial aspect ratio
One-way ANOVA: P=0.0097
P=0.4708
Probability (%)
Median
Mean
Control: 1.473 1.707
CerS6ΔPOMC: 1.491 1.710
Mitochondrial aspect ratio
n
P=0.2261
P=0.524
P=0.654
Mitochondrial area (µm2)
One-way ANOVA: P=0.2435
P=0.7493
Control: 0.097 0.116
CerS6ΔPOMC: 0.094 0.113
Mitochondrial area (µm2)

Supplementary Fig. 7: Depletion of CerS6 in POMC neurons does not affect mitochondrial morphology, feeding behavior, and glucose metabolism in HFD-fed female mice.
Related to Fig. 6
a, PCR analysis on genomic DNA for exon 4 deletion of *CerS6* in ARC-containing tissue samples and the rest of the hypothalamus of control and *CerS6*$^{\Delta POMC}$ mice (n=3 mice/group). **b**, BaseScope duplex in situ hybridization using probes targeting *Pomc* (red) and the exon junction 4/5 in the *CerS6* transcript (*CerS6*^{E4E5}), illustrating expression of full-length *CerS6* (bright blue, top), or the exon junction 3/5 (*CerS6*^{E3E5}), illustrating expression of the recombined exon 4-deficient transcript of *CerS6* (bright blue, bottom) in the hypothalamus of *CerS6*$^{\Delta POMC}$ mice (green) and controls (white); zoom images depict the ventromedial nucleus (VMH) and arcuate nucleus (ARC) of the hypothalamus; blue denotes nuclei stained by haematoxylin; dashed squares indicate zoom range; black arrows depict cells coexpressing *Pomc* and the respective *CerS*6 transcript and yellow arrow depicts a *Pomc*-negative cell with expression of the exon 4-deficient transcript in the ARC of *CerS6*$^{\Delta POMC}$ mice; 3V: third ventricle; scale bars: 150 µm for overview images, 50 µm for zoom images. **c-n**, Analysis of HFD-fed control (white) and *CerS6*$^{\Delta POMC}$ (green) female mice. **c**, Body weight development of mice during HFD feeding (n=10-28 mice/group and week). **d**, Body fat content relative to body weight measured by micro-CT analysis (n=17vs.22 mice). **e**, Fat-pad weights of gonadal white adipose tissue (gWAT), subcutaneous WAT (sWAT), and brown adipose tissue (BAT) relative to body weight (n=5vs.9 mice). **f,** Average energy expenditure over 24 hr normalized for body weight (n=5vs.9 mice). **g**, Absolute food intake over 24 hr (n=5vs.9 mice). **h,** Cumulative food intake after refeeding following a 16 hr fasting period (grey background indicates dark phase) and quantification of absolute food intake (FI) during the dark phase after refeeding (n=5vs.9 mice). **i,** Insulin tolerance test and area under the curve (AUC) for each mouse (n=22vs.28 mice). **j, k,** Glucose tolerance test following a 6 hr (**j**) or 16 hr (**k**) fasting period and AUCs (n=22vs.28 mice). **l**, Representative transmission electron micrographs of mitochondria in ARC POMC neurons of control female mice fed a normal chow (NCD, left) or high-fat diet (HFD, middle), and HFD-fed *CerS6*$^{\Delta POMC}$ mice (right); scale bars: 1 µm. **m,** Violin plots of log2-transformed mitochondrial aspect ratios (n=221-843 mitochondria/group from 5-6 POMC neurons/mouse, 1-3 mice/group) and cumulative distribution function (probability plot) of mitochondrial aspect ratios in HFD-fed *CerS6*$^{\Delta POMC}$ mice and controls (n=774-843 mitochondria/group from 5-6 POMC neurons/mouse, 3 mice/group). **n,** Violin plots of mitochondrial area (n=221-843 mitochondria/group from 5-6 POMC neurons/mouse, 1-3 mice/group) and cumulative distribution function (probability plot) of mitochondrial area in HFD-fed *CerS6*$^{\Delta POMC}$ mice and control littermates (n=774-843 mitochondria/group from 5-6 POMC neurons/mouse, 3 mice/group). All data in **c**-**n** obtained from female mice. Data in **c**-**g** and longitudinal data in **h**-**k** are represented as mean values ±SEM. Boxplots indicate median ±min/max and include data points of individual mice entering the analysis. Dashed lines in violin plots indicate median and dotted lines indicate the first and third quartile, respectively. *P* values calculated using one-way ANOVA followed by Tukey's multiple comparison test (**m**, **n**), Kolmogorov-Smirnov test (distributions in **m**, **n**), two-way RM ANOVA (longitudinal analysis in **h**, **i**-**k**), or a mixed-effects model (**c**). Source data and further details of statistical analyses are provided as a Source Data file.
