# Supplementary material for: CerS6-dependent ceramide synthesis in hypothalamic neurons promotes ER/mitochondrial stress and impairs glucose homeostasis in obese mice
Source: Nat Commun. 2023 Nov 29;14:7824. doi: 10.1038/s41467-023-42595-7 (PMC10684560; doi:10.1038/s41467-023-42595-7)
Supplement: Supplementary file 3 — Reporting Summary [file 41467_2023_42595_MOESM3_ESM.pdf]

nature portfolio

Corresponding author(s): Jens C. Brüning

Last updated by author(s): Aug 23, 2023

Reporting Summary

Nature Portfolio wishes to improve the reproducibility of the work that we publish. This form provides structure for consistency and transparency in reporting. For further information on Nature Portfolio policies, see our Editorial Policies and the Editorial Policy Checklist.

Statistics

For all statistical analyses, confirm that the following items are present in the figure legend, table legend, main text, or Methods section.

n/a	Confirmed	
☐	☒	The exact sample size (*n*) for each experimental group/condition, given as a discrete number and unit of measurement
☐	☒	A statement on whether measurements were taken from distinct samples or whether the same sample was measured repeatedly
☐	☒	The statistical test(s) used AND whether they are one- or two-sided<br>*Only common tests should be described solely by name; describe more complex techniques in the Methods section.*
☐	☒	A description of all covariates tested
☐	☒	A description of any assumptions or corrections, such as tests of normality and adjustment for multiple comparisons
☐	☒	A full description of the statistical parameters including central tendency (e.g. means) or other basic estimates (e.g. regression coefficient) AND variation (e.g. standard deviation) or associated estimates of uncertainty (e.g. confidence intervals)
☐	☒	For null hypothesis testing, the test statistic (e.g. *F*, *t*, *r*) with confidence intervals, effect sizes, degrees of freedom and *P* value noted<br>*Give P values as exact values whenever suitable.*
☒	☐	For Bayesian analysis, information on the choice of priors and Markov chain Monte Carlo settings
☒	☐	For hierarchical and complex designs, identification of the appropriate level for tests and full reporting of outcomes
☒	☐	Estimates of effect sizes (e.g. Cohen's *d*, Pearson's *r*), indicating how they were calculated

Our web collection on statistics for biologists contains articles on many of the points above.

Software and code

Policy information about availability of computer code

Data collection	Indirect calorimetry: TSE PhenoMaster versions 6.2.5 and above<br>CT imaging: IVIS LivingImage Software (version 4.3.1)<br>qPCR analysis: QuantStudio Real-Time PCR software (version 1.7.1)<br>ELISA: Absorption spectroscopy was conducted using the SoftMax pro 6.3 software (Molecular Devices)<br>RNA concentration was measured with software ND-1000 (version 3.8.1)<br>Seahorse analysis: Wave 2.4.1 (Agilent)<br>Imaging of Western blots: FusionCapt Advance (Vilber)<br>Flow cytometry: MACSQuantify (version 2.13.1, Miltenyi Biotec)<br>Microscopy: Confocal images were acquired using a Leica SP8 microscope operated by LasX software (Leica)<br>Brightfield images were acquired using Axio Imager 2 microscope operated by ZEN2 (blue edition; Carl Zeiss Microscopy GmbH).<br>TEM images of N43/5 cells were acquired using software DigitalMicrograph (Gatan, version 3.32.2403.0)<br>Sphingolipidomics: QTRAP 6500 mass spectrometer was used operated by Analyst 1.6.3 (SCIEX) or Q-Exactive HRMS operated by Xcalibur software (Thermo Scientific)
Data analysis	General data visualization and analysis: Prism 9 (version 9.1.0, GraphPad) and Microsoft Excel (version 16.69.1)<br>ANCOVA analysis for energy expenditure of CerS6ΔNkx2.1 mice and controls: CalR Version 1.3 (https://calrapp.org/)<br>Analysis of body composition from CT images: Vinci software package (version 4.61.0)<br><br>Densitometric analysis and image quantification: ImageJ (Fiji) (version 2.9.0/1.53p13)

Sphingolipidomic analysis: MultiQuant 3.0.3 (SCIEX) and TraceFinder 5.1 (Thermo Scientific)
Seahorse analysis: Multi-File Seahorse XF Cell Mito Stress Test Report Generator (Agilent)
Flow cytometry: FlowJo software (BD Biosciences)
The HypoMap single cell data were analyzed using basic plotting functions (FeaturePlot, DotPlot) available through the R Seurat package and R. The associated R code can be made available upon request. Relevant software packages used for this analysis:
R (version 4.2.2)
Seurat R package (version 4.3.0)
ggplot2 R package (version 3.4.0)
dplyr R package (version 1.0.10)

For manuscripts utilizing custom algorithms or software that are central to the research but not yet described in published literature, software must be made available to editors and reviewers. We strongly encourage code deposition in a community repository (e.g. GitHub). See the Nature Portfolio guidelines for submitting code & software for further information.

Data

Policy information about availability of data

All manuscripts must include a data availability statement. This statement should provide the following information, where applicable:

- Accession codes, unique identifiers, or web links for publicly available datasets
- A description of any restrictions on data availability
- For clinical datasets or third party data, please ensure that the statement adheres to our policy

Numerical data and uncropped immunoblots are available as Source Data file.
The seurat object containing HypoMap, which is required to reproduce the single-cell data related figures is available at University of Cambridge's Apollo Repository (doi:10.17863/CAM.87955). The R code used to analyze HypoMap single cell sequencing data is available under https://github.com/lsteuernagel/ceramide_paper_hypomap. The custom made semi-automatic ImageJ macros used to quantify pSTAT3 signal intensity from immunohistochemical stainings are available under https://github.com/mrfeldmann/ceramide_paper.

Human research participants

Policy information about studies involving human research participants and Sex and Gender in Research.

Reporting on sex and gender	N/A
Population characteristics	N/A
Recruitment	N/A
Ethics oversight	N/A

Note that full information on the approval of the study protocol must also be provided in the manuscript.

Field-specific reporting

Please select the one below that is the best fit for your research. If you are not sure, read the appropriate sections before making your selection.

[x] Life sciences   [ ] Behavioural & social sciences   [ ] Ecological, evolutionary & environmental sciences

For a reference copy of the document with all sections, see nature.com/documents/nr-reporting-summary-flat.pdf

Life sciences study design

All studies must disclose on these points even when the disclosure is negative.

Sample size	No statistical methods were used to pre-determine sample sizes. Sample sizes were based on own previous results and according to standards in the field. Sample sizes were similar to those reported previously: e.g., doi:10.1016/j.cell.2019.05.008.
Data exclusions	Sphingolipid data was tested for statistically significant outliers using Grubb's test (https://www.graphpad.com/quickcalcs/grubbs1/). Samples were only removed from the dataset if every acyl chain sphingolipid species of one sample was a significant outlier within the group.<br>Unreasonable jumps in food intake higher than 2g/20min, which occured likely due to technical errors during the PhenoMaster recordings, were removed from the dataset.<br>Other data were only excluded if obvious technical issues occured or because of euthanization of animals due to health complications in line with local animal guidelines.
Replication	Results from cultured cell lines are based on experiments that have been independently performed at least 3 times with one or more technical replicates per experiment, as specified in the figure legends. All attempts for replicates were successful.<br>For metabolic phenotyping, every mouse represents a biological replicate (n) and the numbers are mentioned in each figure and/or figure

	legends. Mice were sequentially sampled, no formal replication study was performed. Blood glucose levels for CerS6ΔSF-1, CerS6ΔAgRP, and CerS6ΔPOMC mice were measured in at least two repetitive measurements using two independent glucose monitors.
Randomization	Sex and age-matched mice were allocated to their respective experimental groups based on their genotypes. Control and transgenic mice were littermates of different litters.<br>C57BL/6N mice used for the analysis of hypothalamic CerS expression and sphingolipid content were randomly assigned to the CD- and HFD-fed group.<br>For cellular assays, treatments were assigned in a random manner (e.g., it was random, which out of two culture plates was used for siCerS6- or scrambled treatment).
Blinding	The analysts performing TEM imaging and quantification of mitochondrial shape in POMC neurons as well as those performing sphingolipidomics were blinded for genotype and treatment until conditions were disclosed for data visualization.<br>For other experiments the analysts were blind for group allocation (genotype and treatment). However, no formal blinding practice was applied, since the persons planning and performing the experiments, and processing and analyzing the data were the same.

Behavioural & social sciences study design

All studies must disclose on these points even when the disclosure is negative.

Study description	N/A
Research sample	N/A
Sampling strategy	N/A
Data collection	N/A
Timing	N/A
Data exclusions	N/A
Non-participation	N/A
Randomization	N/A

Ecological, evolutionary & environmental sciences study design

All studies must disclose on these points even when the disclosure is negative.

Study description	N/A
Research sample	N/A
Sampling strategy	N/A
Data collection	N/A
Timing and spatial scale	N/A
Data exclusions	N/A
Reproducibility	N/A
Randomization	N/A
Blinding	N/A

Did the study involve field work? ☐ Yes ☒ No

Reporting for specific materials, systems and methods

We require information from authors about some types of materials, experimental systems and methods used in many studies. Here, indicate whether each material, system or method listed is relevant to your study. If you are not sure if a list item applies to your research, read the appropriate section before selecting a response.

Materials & experimental systems

n/a	Involved in the study
☐	☒ Antibodies
☐	☒ Eukaryotic cell lines
☒	☐ Palaeontology and archaeology
☐	☒ Animals and other organisms
☒	☐ Clinical data
☒	☐ Dual use research of concern

Methods

n/a	Involved in the study
☒	☐ ChIP-seq
☐	☒ Flow cytometry
☒	☐ MRI-based neuroimaging

Antibodies

Antibodies used

Primary antibodies for Western blot analysis:
Mouse monoclonal anti-CerS6 (5H7), (Abnova Cat# H00253782-M01, RRID:AB_489924, dilution 1:1000)
Rabbit monoclonal anti-ATF4 (D4B8), (Cell Signaling Technology Cat# 11815, RRID:AB_2616025, dilution 1:1000)
Rabbit polyclonal anti-Calnexin (575-593), (Millipore Cat#208880, RRID:AB_2069031, dilution 1:5000)
Mouse monoclonal anti-ACTIN (AC-15), (Sigma-Aldrich Cat# A5441, RRID:AB_476744, dilution 1:10000)
Goat polyclonal GRP78/BiP (N-20) (Santa Cruz Biotechnology Cat# sc1050, RRID:AB_631616, dilution 1:1000)

Secondary antibodies for Western blot analysis:
Goat polyclonal anti-rabbit IgG-peroxidase (Sigma-Aldrich Cat# A0545, RRID:AB_257896, dilution 1:2000)
Goat polyclonal anti-mouse IgG-peroxidase (Sigma-Aldrich Cat# A4416, RRID:AB_258167, dilution 1:2000)
Mouse monoclonal anti-goat/sheep IgG-peroxidase (GT-34), (Sigma-Aldrich Cat# A9452, RRID:AB_258449, dilution 1:2000)

Primary antibodies for flow-cytometry:
Brilliant Violet 421™ anti-mouse/human CD11b Antibody, (M1/70), (BioLegend Cat# 101236, RRID:AB_11203704, dilution 1:150)
O4 Antibody, anti-human/mouse/rat, APC (Miltenyi Biotec Cat# 130-119-155, RRID:AB_2751644, dilution 1:100)
ACSA-2 Antibody, anti-mouse, APC, (IH3-18A3), (Miltenyi Biotec Cat# 130-117-535, RRID:AB_2727978, dilution 1:100))

Primary antibodies for immunohistochemistry and transmission electron microscopy:
Proopiomelanocortin Precursor (POMC) (27-52) antibody (Phoenix Pharmaceuticals Cat# H-029-30, RRID:AB_2307442, dilution 1:7500)
Phospho-Stat3 (Tyr705) (D3A7) XP Rabbit mAb antibody (Cell Signaling Technology Cat# 9145, RRID:AB_2491009, dilution 1:100)

Secondary anitbodies for immunohistochemistry and transmission electron microscopy:
Anti-Rabbit IgG (Goat) antibody (MAb Technologies Cat# NEF812001EA, RRID:AB_2571640, dilution 1:100)
Donkey Anti-Rabbit IgG (H+L) Antibody, Alexa Fluor 488 Conjugated, (A-21206), (Molecular Probes Cat# A-21206, RRID:AB_2535792, dilution 1:1000)
Biotin-SP-AffiniPure Donkey Anti-Rabbit IgG (H+L) antibody (Jackson ImmunoResearch Labs Cat# 711-065-152, RRID:AB_2340593, dilution 1:250)

Validation

The CerS6 antibody was validated for Western blot analysis with CerS6-deficient mouse tissue and cultured cells in-house before (doi:10.1016/j.cell.2019.05.008).

For all other antibodies please refer to the manufacturer's description of the antibodies:

Mouse monoclonal anti-CerS6 (5H7) (Abnova Cat# H00253782-M01, RRID:AB_489924): https://www.abnova.com/protocol_pdf/DS_H00253782-M01.pdf

Rabbit monoclonal anti-ATF4 (D4B8) (Cell Signaling Technology Cat# 11815, RRID:AB_2616025): https://www.cellsignal.com/datasheet.jsp?productId=11815&images=1&size=A4

Rabbit polyclonal anti-Calnexin (575-593), (Millipore Cat#208880, RRID:AB_2069031): https://www.merckmillipore.com/DE/de/product/Anti-Calnexin-C-Terminal-575-593-Rabbit-pAb,EMD_BIO-208880#anchor_PDS

Mouse monoclonal anti-ACTIN (AC-15), (Sigma-Aldrich Cat# A5441, RRID:AB_476744): https://www.sigmaaldrich.com/specification-sheets/141/510/A5441-BULK________SIGMA____.pdf

Goat polyclonal GRP78/BiP (N-20) (Santa Cruz Biotechnology Cat# sc1050, RRID:AB_631616): https://datasheets.scbt.com/sc-1050.pdf

Goat polyclonal anti-rabbit IgG-peroxidase (Sigma-Aldrich Cat# A0545, RRID:AB_257896): https://www.sigmaaldrich.com/specification-sheets/327/941/A0545-BULK________SIGMA____.pdf

Goat polyclonal anti-mouse IgG-peroxidase (Sigma-Aldrich Cat# A4416, RRID:AB_258167): https://www.sigmaaldrich.com/specification-sheets/200/206/A4416-BULK________SIGMA____.pdf

Mouse monoclonal anti-goat/sheep IgG-peroxidase, clone GT-34 (Sigma-Aldrich Cat# A9452, RRID:AB_258449): https://www.sigmaaldrich.com/specification-sheets/393/427/A9452-BULK________SIGMA____.pdf

Brilliant Violet 421™ anti-mouse/human CD11b Antibody, (M1/70), (BioLegend Cat# 101236, RRID:AB_11203704): https://d1spbj2x7qk4bg.cloudfront.net/Default.aspx?ID=10267&pdf=true&displayInline=true&ProductID=7163&leftRightMargin=15&topBottomMargin=15&filename=Brilliant%20Violet%20421™%20anti-mouse/human%20CD11b%20Antibody.pdf&v=20230524063105

O4 Antibody, anti-human/mouse/rat, APC (Miltenyi Biotec Cat# 130-119-155, RRID:AB_2751644): https://static.miltenyibiotec.com/asset/150655405641/document_og3ujsnjot4jf5l429vn663b0r?content-disposition=inline

ACSA-2 Antibody, anti-mouse, APC, (IH3-18A3), (Miltenyi Biotec Cat# 130-117-535, RRID:AB_2727978): https://static.miltenyibiotec.com/asset/150655405641/document_ovasi59abl6oj5cjb8dtm2ko3m?content-disposition=inline

Proopiomelanocortin Precursor (POMC) (27-52) antibody (Phoenix Pharmaceuticals Cat# H-029-30, RRID:AB_2307442): https://www.phoenixpeptide.com/products/view/Antibodies/H-029-30

Phospho-Stat3 (Tyr705) (D3A7) XP Rabbit mAb antibody (Cell Signaling Technology Cat# 9145, RRID:AB_2491009): https://www.cellsignal.com/datasheet.jsp?productId=9145&images=1&size=A4

Anti-Rabbit IgG (Goat) antibody (MAb Technologies Cat# NEF812001EA, RRID:AB_2571640): https://stella.mabtech.com/sites/default/files/product_datasheets/3310-7-1000.pdf

Donkey Anti-Rabbit IgG (H+L) Antibody, Alexa Fluor 488 Conjugated, (A-21206) (Molecular Probes Cat# A-21206, RRID:AB_2535792): https://www.thermofisher.com/order/genome-database/dataSheetPdf?producttype=antibody&productsubtype=antibody_secondary&productId=A-21206&version=320

Biotin-SP-AffiniPure Donkey Anti-Rabbit IgG (H+L) antibody (Jackson ImmunoResearch Labs Cat# 711-065-152, RRID:AB_2340593): https://www.jacksonimmuno.com/catalog/products/711-065-152

Mouse monoclonal anti-goat/sheep IgG-peroxidase, (GT-34), (Sigma-Aldrich Cat# A9452, RRID:AB_258449): https://www.sigmaaldrich.com/specification-sheets/298/716/SAB4200804-BULK___SIGMA____.pdf

Eukaryotic cell lines

Policy information about cell lines and Sex and Gender in Research

Cell line source(s)	For in vitro experiments we employed the immortalized embryonic mouse hypothalamus cell line N43/5 (mHypoE-N43/5), which was derived originally from a POMC-positive fetal hypothalamic neuron as described previously by D.D. Belsham (doi:10.1677/JOE-06-0080).
Authentication	The cell line has not been authenticated in the current study.
Mycoplasma contamination	The cell line was tested negative for mycoplasma contamination.
Commonly misidentified lines (See ICLAC register)	No commonly misidentifed cell lines were used in this study.

Animals and other research organisms

Policy information about studies involving animals; ARRIVE guidelines recommended for reporting animal research, and Sex and Gender in Research

Laboratory animals	C57BL/6N male mice (strain code: 027) were purchased from Charles River laboratories at 4 weeks of age.<br>db/db male mice and misty controls were purchased from The Jackson Laboratory.<br>CerS6Δ/Δ mice (doi:10.1016/j.cmet.2014.08.002) were obtained from in-house breedings.<br>For hypothalamic ablation, CerS1fl/fl (doi:10.1016/j.celrep.2018.12.031) and CerS6fl/fl mice (doi:10.1016/j.cmet.2014.08.002) were bred to mice expressing the Nkx2.1-Cre transgene (doi:10.1002/cne.21529). CerS6fl/fl animals were also bred to previously described SF-1-Cre (doi:10.1016/j.neuron.2005.12.021), AgRP-IRES-Cre (doi:10.1038/nn.2167), and POMC-Cre-expressing mice (doi:10.1016/j.neuron.2004.06.004). Mice between 4-43 wk were used for experiments. Specific age of mice for each experiment is described in Methods.<br>For detailed information on mouse husbandry and animal care, we refer the reader to the Methods section of the manuscript.
Wild animals	No wild animals were used in this study.
Reporting on sex	Male and female mice were analyzed separately. Sex of mice is specified in the figure legends and in the body weight graphs.
Field-collected samples	No field-collected samples were used in this study.

Ethics oversight	All animal procedures were conducted in compliance with protocols approved by local government authorities (Bezirksregierung Köln).<br>Permission to maintain and breed mice as well as for all experimental protocols in this study was issued by the Department for Environment and Consumer Protection - Veterinary Section, Köln, North Rhine-Westphalia, Germany .

Note that full information on the approval of the study protocol must also be provided in the manuscript.

Clinical data

Policy information about clinical studies

All manuscripts should comply with the ICMJEguidelines for publication of clinical research and a completedCONSORT checklist must be included with all submissions.

Clinical trial registration	N/A
Study protocol	N/A
Data collection	N/A
Outcomes	N/A

Dual use research of concern

Policy information about dual use research of concern

Hazards

Could the accidental, deliberate or reckless misuse of agents or technologies generated in the work, or the application of information presented in the manuscript, pose a threat to:

No	Yes	
☐	☐	Public health
☐	☐	National security
☐	☐	Crops and/or livestock
☐	☐	Ecosystems
☐	☐	Any other significant area

Experiments of concern

Does the work involve any of these experiments of concern:

No	Yes	
☐	☐	Demonstrate how to render a vaccine ineffective
☐	☐	Confer resistance to therapeutically useful antibiotics or antiviral agents
☐	☐	Enhance the virulence of a pathogen or render a nonpathogen virulent
☐	☐	Increase transmissibility of a pathogen
☐	☐	Alter the host range of a pathogen
☐	☐	Enable evasion of diagnostic/detection modalities
☐	☐	Enable the weaponization of a biological agent or toxin
☐	☐	Any other potentially harmful combination of experiments and agents

ChIP-seq

Data deposition

☐ Confirm that both raw and final processed data have been deposited in a public database such as GEO.

☐ Confirm that you have deposited or provided access to graph files (e.g. BED files) for the called peaks.

Data access links<br>*May remain private before publication.*	N/A
Files in database submission	N/A
Genome browser session<br>(e.g. UCSC)	N/A

Methodology

Replicates	N/A
Sequencing depth	N/A
Antibodies	N/A
Peak calling parameters	N/A
Data quality	N/A
Software	N/A

Flow Cytometry

Plots

Confirm that:

- [x] The axis labels state the marker and fluorochrome used (e.g. CD4-FITC).
- [x] The axis scales are clearly visible. Include numbers along axes only for bottom left plot of group (a 'group' is an analysis of identical markers).
- [x] All plots are contour plots with outliers or pseudocolor plots.
- [ ] A numerical value for number of cells or percentage (with statistics) is provided.

Methodology

Sample preparation	Adult mouse hypothalami were dissociated using the Adult Brain Dissociation Kit for mouse and rat (#130-107-677, Miltenyi Biotec) in combination with the gentleMACS Octo Dissociator with Heaters (#130-096-427, Miltenyi Biotec) according to the manufacturer's instructions. Separation of neuronal and non-neuronal cell fractions was performed using the Neuron Isolation Kit for mouse (#130-115-389, Miltenyi Biotec) according to the manufacturer's instructions. For more details we refer to the reador to the Methods section in the manuscript.
Instrument	MACSQuant 10 (Miltenyi Biotec).
Software	MACSQuantify (version 2.13.1, Miltenyi Biotec) and FlowJo software (BD Biosciences).
Cell population abundance	No rare cell populations were investigated.
Gating strategy	Dead cells were excluded based on propidium iodide fluorescence.

- [x] Tick this box to confirm that a figure exemplifying the gating strategy is provided in the Supplementary Information.

Magnetic resonance imaging

Experimental design

Design type	N/A
Design specifications	N/A
Behavioral performance measures	N/A

Acquisition

Imaging type(s)	N/A
Field strength	N/A
Sequence & imaging parameters	N/A
Area of acquisition	N/A

Diffusion MRI ☐ Used ☐ Not used

Preprocessing

Preprocessing software	N/A

Normalization	N/A
Normalization template	N/A
Noise and artifact removal	N/A
Volume censoring	N/A

Statistical modeling & inference

Model type and settings	N/A
Effect(s) tested	N/A

Specify type of analysis: ☐ Whole brain ☐ ROI-based ☐ Both

Statistic type for inference (See [Eklund et al. 2016])	N/A
Correction	N/a

Models & analysis

n/a	Involved in the study
☐	☐ Functional and/or effective connectivity
☐	☐ Graph analysis
☐	☐ Multivariate modeling or predictive analysis

Functional and/or effective connectivity	N/A
Graph analysis	N/A
Multivariate modeling and predictive analysis	N/A
